# Polymeric nanoparticles as an alternative for application of gibberellic acid in sustainable agriculture: a field study

**DOI:** 10.1038/s41598-019-43494-y

**Published:** 2019-05-09

**Authors:** Anderson do Espírito Santo Pereira, Halley Caixeta Oliveira, Leonardo Fernandes Fraceto

**Affiliations:** 1 São Paulo State University (UNESP), Institute of Science and Technology, Sorocaba, Avenida Três de Março, 511, CEP 18087-180 Sorocaba, SP Brazil; 2 Department of Animal and Plant Biology, University of Londrina, PR 445, km 380, CEP 86047-970 Londrina, PR Brazil

**Keywords:** Plant sciences, Nanoscience and technology

## Abstract

Nanocarrier systems for the encapsulation of agrochemicals can contribute to sustainable agriculture, but few nanosystems have been developed for plant growth regulators (PGRs). The present study evaluated the effects of seed priming using alginate/chitosan (*nano*ALG/CS) and chitosan/tripolyphosphate (*nano*CS/TPP) containing GA_3_ on the growth and productivity of *Solanum lycopersicum* cultivated under field conditions. The results demonstrated that nanocarrier systems could improve fruit production, with the productivity increasing almost 4-fold using *nano*ALG/CS-GA_3_. This pioneering study demonstrates the potential of nanocarrier systems with PGRs for applications in agriculture.

## Introduction

Sustainability, food safety, increased production, and reduction of environmental contaminants are among the greatest challenges of agriculture, faced with problems such as decreased space, depletion of natural resources, and climate change^1,2^. Consequently, new policies, management strategies, and technologies are needed to ensure the supply of food for a population that will reach 9 billion by 2050^3^.

The green agricultural revolution of the 1960s created a dependency on the use of pesticides, resulting in serious impacts including environmental harm, health problems in agriculturalists, and low agricultural sustainability. This has led to the need for new approaches and research in order to shift agricultural practices towards greater sustainability^4^.

Technologies such as the development of plant hybrids, chemical synthesis, and biotechnology have benefited the agricultural sector in recent years. Nanotechnology can contribute to greater agricultural sustainability^5^ and has attracted attention due to its potential for use in a wide range of applications^6^. It can assist in reducing the concentrations of agrochemicals used in the field, while at the same time improving the quality and productivity of agricultural crops^5^. Other advantages include better soil quality, decreased water contamination, and less risk to consumers and agricultural workers^6^.

Among their many possible applications, nanoparticulate systems can be used for loading with bioactive compounds, as a more sustainable alternative, compared to conventional methods. These systems can promote sustained release, convey active agents to specific targets, and enhance bioavailability at the target organism^2,7^.

In agricultural applications, nanoparticulate systems have shown promise for increasing the biological activity and reducing the toxicity of herbicides, fungicides, and insecticides^2,6,8^. However, it is essential that prior to the use of these systems, studies must be carried out to evaluate their impacts in the environment and on human health^8^. All these systems can indirectly help to increase productivity when they are used for pest control, but few nanocarrier systems have been explored for use with plant growth regulators.

Plant growth regulators (PGRs) are a class of compounds with extremely important roles. These plant hormones, or molecules that alter plant hormonal homeostasis and signaling, can be applied to crops to enhance plant development, increase production, improve the visual and nutritional aspects of food, and increase the storage time or shelf life^9^. An example is gibberellic acid (GA_3_), which is one of the PGRs most extensively used for a variety of crops^10^. One of its uses, for example, is the breaking of seed dormancy, which involves morphological and physiological mechanisms. Gibberellins stimulate the synthesis of hydrolases, especially α-amylases, which render the endosperm reserves available to the embryo^11^.

In agriculture, seed vigor is essential for germination and the rapid growth of robust seedlings, leading to satisfactory yields^12^. Diverse types of seed pretreatments employing a limited amount of water (collectively called seed priming) are used to improve the metabolic activity prior to germination, such as osmotic conditioning (hydration followed by drying) and hormone priming^13,14^. The advantages of these treatments include increased emergence, enhanced ability to compete with weeds for resources, better development under stress conditions, improved resistance to pathogens, and increased yields^14^. Many studies have demonstrated the beneficial effects of seed pretreatment with GA_3_ in different crops, including increased productivity of *Triticum aestivum* under drought and unstressed conditions^15^, increased water uptake by seeds of *Beta vulgaris* under saline conditions^16^, shorter emergence time of *Glycine max* seedlings^17^, and increased plant weight and yield of *Helianthus annuus*^18^.

However, it is important to apply GA_3_ at a suitable concentration and at the correct time, in order to avoid adverse effects on plant development and crop yield^19^. In a study with *Leymus chinensis*, Ma *et al*.^20^ reported the biphasic effect of GA_3_ seed priming on development of the grass. The aboveground biomass increased up to the optimum GA_3_ concentration (50 µM), above which the effect of the hormone decreased. In this way, the use of nanocarrier systems for GA_3_ could enable slow and sustained release of the active agent, avoiding the attainment of supra-optimal levels. In addition, the nanoencapsulation could improve the solubility of GA_3_ and protect it from degradation^21–23^.

There have been few studies concerning the development of nanocarrier systems for PGRs such as brassinosteroids, *O*-naphthyl acetyl, and GA_3_ ^21–24^. According to Yang *et al*.^22^, an inclusion complex of GA_3_ with cyclodextrins enhanced the chemical stability of the hormone and increased the growth of mung bean and cucumber seedlings. In studies using alginate/chitosan (ALG/CS) and chitosan/tripolyphosphate (CS/TPP) nanocarrier systems containing GA_3_, it was shown that the treatment of seeds led to beneficial effects in the development of *Phaseolus vulgaris* plants. After seven days, the seedlings showed increases of root development, leaf area, and photosynthetic pigments, compared to use of the hormone. The results demonstrated that these systems were able to improve the vigor of seeds of *P. vulgaris*^21,23^. However, these studies did not investigate the potential of the nanocarrier systems under field conditions.

This study describes the results obtained for two chitosan-based nanoparticle systems loaded with GA_3_. Seeds of *Solanum lycopersicum* var. cerasiforme were treated with chitosan/tripolyphosphate and alginate/chitosan nanoparticles containing GA_3_ (*nano*CS/TPP-GA_3_ and *nano*ALG/CS-GA_3_, respectively), and evaluation was made of the effects on plant growth and fruit production under field conditions. It should be highlighted that there have been no previous studies reported in the literature that have investigated the growth of plants following application of a natural plant growth regulator associated with biodegradable polymer nanoparticles prepared without the use of organic solvents. These new systems offer benefits for use in sustainable agricultural production practices.

## Materials and Methods

### Materials

Chitosan (MW 27 kDa), sodium alginate, and the GA_3_ plant hormone were obtained from Sigma-Aldrich. Seeds of *Solanum lycopersicum* var. cerasiforme were removed from fruits purchased in a local market in São Paulo (Brazil) and were dried at room temperature.

### Preparation of nanoparticles

The CS nanoparticle formulations were prepared with a final GA_3_ concentration of 0.05 mg/mL. The formulations were characterized in an earlier study by dynamic light scattering (DLS), nanoparticle tracking analyses (NTA), and atomic force microscopy (AFM)^21^. The *nano*ALG/CS nanoparticles presented a size of 450 nm, PDI of 0.3, and zeta potential of −29 mV, while the *nano*CS/TPP particles presented a size of 195 nm, PDI of 0.3, and zeta potential of +27 mV^21^. The GA_3_ encapsulation efficiencies were 100% (*nano*ALG/CS) and 90% (*nano*CS/TPP)^21^.

#### *Nano*CS/TPP-GA_3_

An aqueous solution of 10 mL of CS (0.2%, pH 4.5) containing 0.6% acetic acid was kept under vigorous stirring. GA_3_ was added, with agitation until complete dissolution, followed by slow addition of 6 mL of TPP solution (0.1%, pH 4.5 at 4 °C), using a pipette. The final formulation was a colloidal dispersion that was kept under stirring for a further 20 minutes.

#### *Nano*ALG/CS-GA_3_

Firstly, 59 mL of an aqueous solution of ALG (0.063%, pH 4.9) was kept under stirring and 3.75 mL of an aqueous solution of CaCl_2_ (50 mM) was added over a period of 1 hour, using a peristaltic pump. Subsequently, GA_3_ was added, with agitation until complete dissolution. The ALG/CaCl_2_/GA_3_ solution was kept under agitation and 12.5 mL of an aqueous solution of CS (0.07%, pH 4.5) was added over a period of 1.5 hours, using a peristaltic pump, resulting in the formation of a colloidal dispersion.

### Treatment of the seeds

The seeds were pretreated using GA_3_, *nano*CS/TPP-GA_3_, and *nano*ALG/CS-GA_3_ at concentrations of 0.05 mg/mL (stock formulation) and dilutions of 0.005 mg/mL (1:10) and 0.0005 mg/mL (1:100). The controls were distilled water and aqueous solutions of TPP (0.03%, m/v), CS (0.12%, m/v), and ALG (4.9 × 10^−4^%, m/v) (these concentrations were the same as those of the final colloidal dispersions of nanoparticles). Dilutions of the *nano*ALG/CS-GA_3_ nanoparticles were performed with a solution of CaCl_2_ (2.5 mM), while the CS/TPP nanoparticles were diluted with a solution of TPP (0.03%, m/v). These concentrations were employed as controls and for dilution of the nanoparticles, since the same concentrations of CaCl_2_ and TPP were used for the nanoparticles in the colloidal dispersions.

For the seed priming process, 50 seeds per treatment were placed in 50 mL Erlenmeyer flasks containing 10 mL of each solution, with agitation overnight for 12 hours, in the dark, at room temperature. After the treatment period, the seeds were sown in germination trays containing Carolina substrate (composed of peat, vermiculite, limestone, and agricultural gypsum) and left for 30 days in a greenhouse. Twelve seedlings were removed for obtaining data on the initial growth, considering the root and shoot lengths (cm) and dry weight (mg).

### Transfer to the soil

After 30 days, 12 seedlings from each treatment were transferred to the soil and kept for a further 90 days in the greenhouse at an average temperature of 25–35 °C. The field experiment was conducted in the city of São Miguel Arcanjo (São Paulo State, Brazil). The region is located in a subtropical zone and during the experiment, the temperature ranged from 20 to 30 °C, with relative humidity of around 40%. The soil of the region is classified as clayey. The seedlings were planted in soil previously prepared with organic fertilizer, with a spacing of 20 cm between the plants. After 90 days, measurements were made of the shoot length (m), shoot fresh and dry masses (g), number of fruits per plant, and fruit weight (g). The dry weight was determined after keeping the plants in an oven at 60 °C for 7 days. The number of fruits considered the total produced by each plant and the fruit weight was obtained by weighing 50 fresh tomatoes.

### Productivity estimation

The productivity was calculated as the fruit fresh weight produced per hectare, considering that each plant was cultivated in an area of 0.5 m^2^.

### Efficacy of the biological effect

The efficacies of the nanocarrier systems were evaluated by comparing the results obtained for the nanoparticles containing GA_3_ and GA_3_, at equivalent concentrations. The data were treated according to Eq. 1.

1 $$B{E}_{NPS}( \% )=[(\frac{NP{s}_{(effect)}}{G{A}_{3(effect)}})\times 100]-100$$

where, BE_NPS_ is the biological effect caused by the treatment with the nanoparticles, NPs_(effect)_ is the result obtained for the evaluated parameters after treatment with the nanocarrier system containing GA_3_, and GA_3(effect)_ is the result obtained using GA_3_. The results are expressed as % biological efficacy.

### Statistical analysis

The data obtained were treated by calculation of the means and standard deviations (n = 12), followed by analysis of variance (ANOVA) with Tukey’s post-hoc test (p < 0.05), using GraphPad Prism 5.01 software.

## Results and Discussion

### Initial development (30 days)

Seed pretreatments are used to improve the synchrony of germination and increase seed vigor due to the activation of metabolic processes^25^. To this end, nanocarrier systems can provide a mean for increasing the effectiveness of the treatment and promoting greater seed vigor. In all cases, the average germination rate was 90% in a total of 50 seeds, with no differences among the treatments (results not shown).

**Figure 1:**
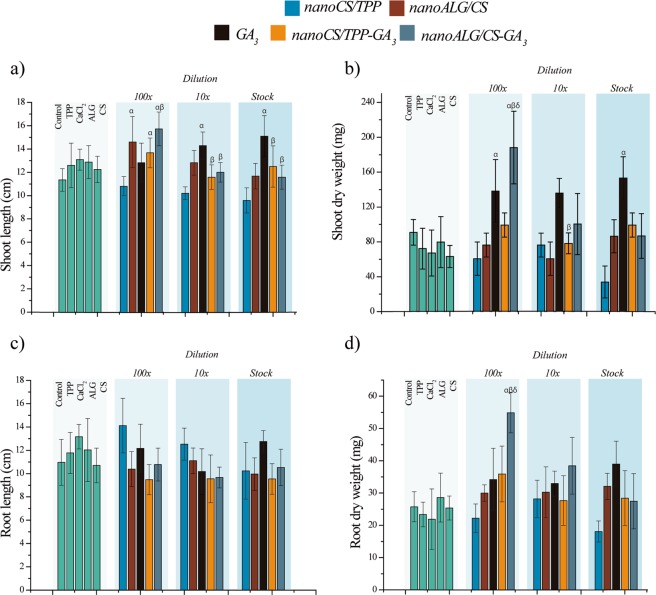
Initial seedling growth 30 days after sowing: (**a**) shoot length (cm); (**b**) shoot dry weight (mg); (**c**) root length (cm); (**d**) root dry weight (mg). Seed treatments using the stock solution and dilutions of 1:10 and 1:100 (v/v), equivalent to concentrations of 0.05, 0.005, and 0.0005 mg/mL, respectively. Data shown as means and standard deviations (n = 12). Statistical analyses using one-way ANOVA with Tukey’s post-hoc test (significance level of p < 0.05), where α, β, and δ indicate significant differences relative to the control (water), treatment with GA_3_, and treatment with *nano*CS/TPP-GA_3_, respectively.

Figure 1 shows the effects of the seed treatments on seedling development, 30 days after germination. At the 100-fold dilution, the *nano*ALG/CS-GA_3_ formulation was able to increase the shoot length and dry weight by 38 and 107%, respectively, compared to the control (Fig. 1a,b). These parameters of plants whose seeds were treated with *nano*ALG/CS-GA_3_ were also higher than those from seeds treated using GA_3_ at the same concentration. For GA_3_ at 100-fold dilution, the only significant effect, compared to the control, was a 52% increase of shoot dry weight (Fig. 1b). The use of *nano*CS/TPP carriers loaded with GA_3_ resulted only in a 32% increase in shoot length, which did not differ from the treatment with GA_3_ (Fig. 1a). The use of the *nano*ALG/CS carrier alone (without GA_3_), diluted 100-fold, also resulted in a 29.3% increase in shoot length, compared to the control (water) (Fig. 1a).

At higher concentrations, using the stock formulation and the 10-fold dilution, treatment with GA_3_ increased shoot length by 33.7 and 26.5%, respectively, compared to the control (water), with the effects being significantly greater than those achieved for both nanoparticles loaded with GA_3_ at the same concentrations (Fig. 1a). In addition, the stock formulation of GA_3_ led to higher shoot dry weight, compared to the control (Fig. 1b). None of the analyzed parameters was affected by the treatments with the nanoformulations at higher concentrations (stock formulation and 10-fold dilution) (Fig. 1a,b).

The different treatments showed no effects on main root length (Fig. 1c). However, treatment using *nano*ALG/CS-GA_3_ diluted 100-fold resulted in a 113% increase in root dry weight, while use of GA_3_ did not affect this parameter, demonstrating a clear effect of this carrier system loaded with GA_3_ (Fig. 1d). The results considered the entire root weight (including the main and lateral roots).

Overall, these results demonstrated that the concentration was an important factor that influenced the biological effects of the different formulations. The effects of the nanoformulations on early growth of the seedlings were observed only at the lowest concentration, whereas the effects of GA_3_ occurred when the stock formulation and the 10-fold dilution were used. This observation could be explained by the direct contact of GA_3_ present in the medium with the seeds, which did not occur for the GA_3_ in the nanoparticles, since the active agent was encapsulated within the polymeric wall and was therefore released more slowly during the seed priming process (which lasted 12 hours). In previous studies, it was shown that the total release of GA_3_ occurred within 24 hours in water (under sink dilution conditions), with factors such as pH and temperature affecting the release profile^21^. However, the behavior could be different after seed exposure, since interaction with the seed components could alter the release profile. Furthermore, some nanoparticles could have remained attached to the seed surface, maintaining the delivery of GA_3_ to the seeds after the priming process. Possible uptake of the nanoparticles by the seeds and subsequent release within the plant tissues should also be considered. The uptake of nanomaterials by seeds was reported by Khodakovskaya *et al*.^26^, who found that carbon nanotubes were able to penetrate tomato seeds, resulting in increased seed germination and plant development. Our earlier work showed that use of these nanocarrier systems led to greater vigor of seeds of *Phaseolus vulgaris*, resulting in increased rates of germination^21^. In the present study, the tomato seeds germinated successfully and rapidly, even in the case of the control seeds treated with water. Hence, there were no observable differences in the effects of the different treatments. Further studies should therefore be undertaken to test the effects of the nanoformulations on seeds presenting dormancy or low germination rates, which could lead to novel applications of these nanoformulations for obtaining faster and more uniform emergence.

In the initial evaluation, it was demonstrated that the most effective treatment for plant development was *nano*ALG/CS-GA_3_ used at a 100-fold dilution, which increased the shoot and root dry weights. The *nano*CS/TPP-GA_3_ formulation showed relatively low biological activity during the initial growth of the tomato seedlings, even when compared with GA_3_.

Previous studies have shown that different nanomaterials may have agricultural applications. For example, metallic or carbon nanomaterials have been used to increase the germination and initial development of plants, aiming at future applications as nutritional supplements or stimulants^7,27^. The uptake of nanoparticles by plants can lead to their accumulation in different plant tissues, with potential undesirable effects in the short or long term^27^.

One of the great advantages of using biodegradable polymer systems, instead of the systems mentioned above, is that they can be used in the metabolism of living organisms. An important point is that neither *nano*CS/TPP-GA_3_ nor *nano*ALG/CS-GA_3_ showed any phytotoxic effects during the stages of germination and initial growth of the tomato plants, even at high concentrations.

The nanoparticles used in this study were mainly composed of CS and ALG, which are edible nontoxic polymers employed in various pharmaceutical and agricultural applications, and can avoid adverse effects such as bioaccumulation. Another point is that the production of the nanoparticles did not involve the use of organic solvents, so the systems were produced entirely from biodegradable materials and were loaded with an active agent that is a plant metabolite. Therefore, biopolymer-based formulations are suitable for use in the development of more sustainable agricultural practices.

Other polymers such as poly(lactic-*co*-glycolic acid) (PLGA), poly(epsilon-caprolactone) (PCL), and zein have shown potential for use in the development of nano-based delivery systems for plants^28–30^. It has been found that PLGA nanoparticles can be taken up by roots and by stomata in the leaves, and can be transported through the vascular system^29^. These nanoparticles can adhere strongly to the outer side of the cell wall, due to electrostatic interactions, and were found to be able to enter grapevine cells by clathrin-dependent or clathrin-independent endocytosis^30^. In the case of PCL nanoparticles, entry into the mesophyll can also occur through stomatal pores and hydathodes^31^. Prasad *et al*.^32^ reported that zein nanoparticles were internalized by the roots and were transported to different plant organs. Since such nanoparticles can penetrate the plant tissue, they may be transported within the roots by apoplastic and symplastic pathways, which are involved in the uptake of water and nutrients^27^.

In the case of seeds, the surfaces possess pores whose selective permeability restricts the uptake of solids or particles. However, intracellular spaces in the parenchyma tissue may be filled with liquid media that facilitate the transfer of nutrients or particles to the embryo, and another mechanism of particle entry into seeds is through aquaporins^27^.

### Plant development (120 days)

After 120 days, the plants were collected and analyses were made of the shoot length, the shoot dry weight, and the number and weight of the fruits.

**Figure 2:**
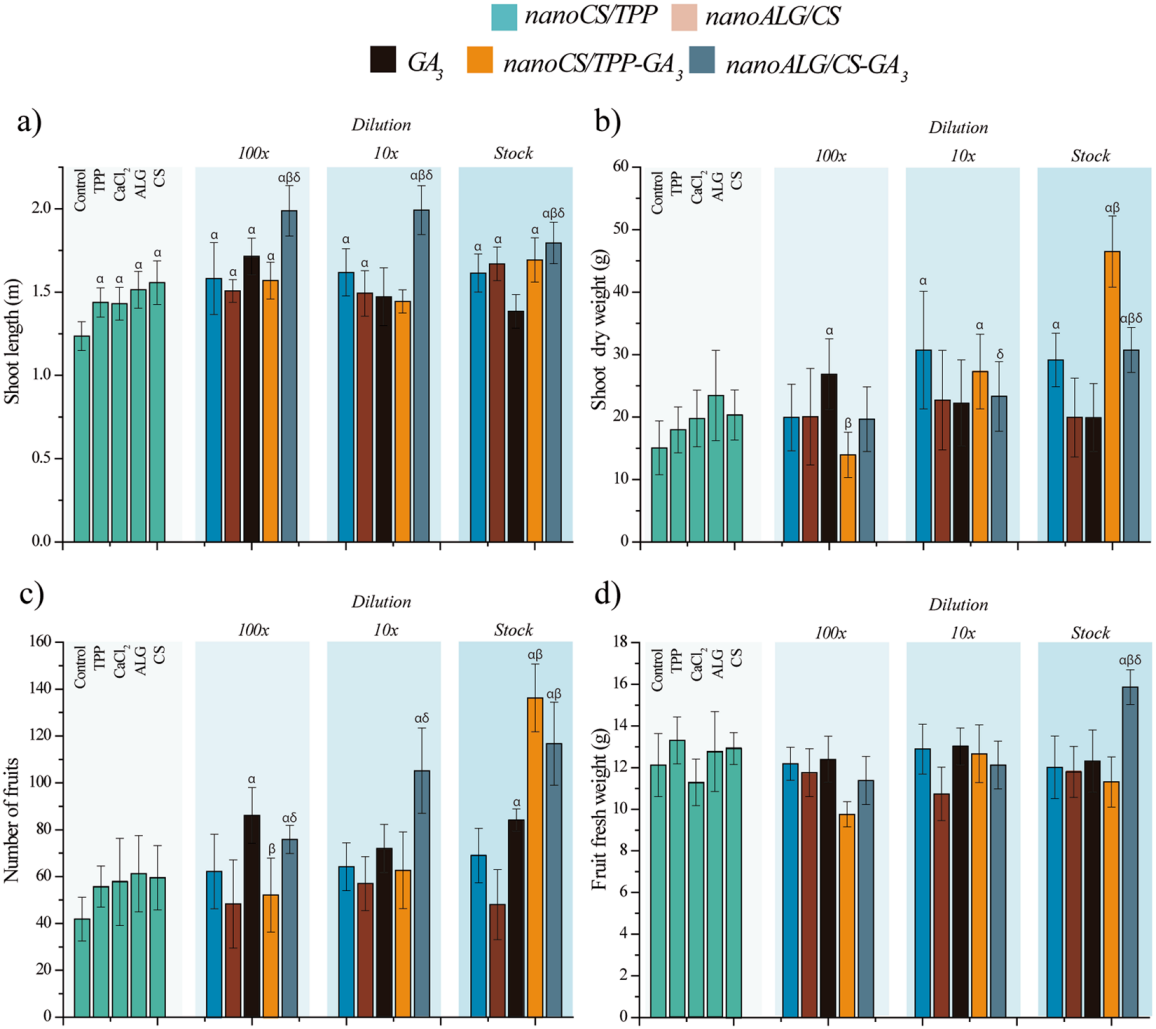
Evaluation of plant development 120 days after sowing: (**a**) length of the aerial part (cm); (**b**) dry weight of the aerial part (g); (**c**) number of fruits per plant; (**d**) fruit fresh weight (g). Seed treatments using the stock solution and dilutions of 1:10 and 1:100 (v/v), equivalent to concentrations of 0.05, 0.005, and 0.0005 mg/mL, respectively. Data presented as means and standard deviations (n = 12). Statistical analyses using one-way ANOVA with Tukey’s post-hoc test (significance level of p < 0.05), where α, β, and δ indicate significant differences relative to the control (water), treatment with GA_3_, and treatment with *nano*CS/TPP-GA_3_, respectively.

The shoot length of plants from seeds treated with *nano*ALG/CS-GA_3_ was greater than for plants from seeds treated with GA_3_ (Fig. 2a). For the treatments with the stock formulation and the 10- and 100-fold dilutions, the use of *nano*ALG/CS-GA_3_ resulted in increases of 45, 63, and 61%, respectively, relative to the water control, compared to increases of 11.6, 18.9, and 40.32% obtained using GA_3_. The shoot length was also affected by the treatments with CaCl_2_ (16.3% increase), TPP (17.14%), ALG (25.3%), CS (26.5%), and nanoparticles without GA_3_.

Several factors could explain the observed effects. For example, the Ca^2+^ ion is a messenger species involved in many cell signaling processes related to stress and hormonal regulation in plants. For this reason, treatment of seeds with CaCl_2_ has been reported to alleviate the negative effects of abiotic stresses and enhance plant growth^33^. Biopolymers such as CS can form thin semi-permeable films around seeds, acting to maintain humidity and consequently promote germination. Polymers can also alter seed metabolism, improving the mechanisms of defense against pathogens and increasing the vigor of seedlings^34^.

In the present case, improved effects were observed for the treatments using *nano*ALG/CS-GA_3_, reflecting the greater efficiency achieved when the active agent was encapsulated. In addition, previous studies have found that the chitosan polymer mixed in soil can enhance plant growth and act as a growth stimulator^35–37^. Some nanomaterials can influence seed vigor, which has been attributed to the generation of increased numbers of pores on the seed surface, resulting in greater water penetration and uptake of nutrients from the soil^26,38^.

The treatments with *nano*CS/TPP-GA_3_ using the stock formulation and the 10-fold dilution increased the shoot dry weight by 208 and 81%, respectively (Fig. 2b). These increases were significantly different to those achieved with GA_3_, for which the weight increases were only 32 and 47%, compared to the control (water). These results indicated greater long-term biological efficacy of *nano*CS/TPP-GA_3_, compared to GA_3_. In terms of fruit production (Fig. 2c), treatments using the nanoparticle stock formulations significantly increased the number of fruits, with increases of 225.5% (*nano*CS/TPP-GA_3_) and 178.8% (*nano*ALG/CS-GA_3_), relative to the control (water), compared to an increase of 101% for the treatment with GA_3_.

When the concentration of *nano*CS/TPP-GA_3_ was reduced, there were no differences in the number of fruits per plant, relative to the control (water). However, at 10-fold dilution, the *nano*ALG/CS-GA_3_ system maintained high fruit production, with an increase of 151.2%, compared to 72.0% for GA_3_. Increased fruit production was also observed using the formulations at 100-fold dilution, with increases of 81.2% for *nano*ALG/CS-GA_3_ and 105% for GA_3_, relative to the control (water). Hence, both formulations were still able to increase fruit production, even after substantial dilution. However, *nano*CS/TPP-GA_3_ at 100-fold dilution did not induce an increase of fruit number. The results for fruit production are summarized in Table 1.

**Table 1:** Summary of the results for tomato fruit production (shown in Fig. 1).

Fruit production			
	0.5 mg/mL (stock solution)	0.05 mg/mL (10-fold dilution)	0.005 mg/mL (100-fold dilution)
GA_3_	101%	72%	105%
*nano*CS/TPP-GA_3_	225%	49%	24%
*nano*ALG/CS-GA_3_	178%	151%	81%

The data are given as % production, compared to the control (seed priming with water).

In terms of average fruit weight, most of the treatments presented no significant differences relative to the control (water), although the use of *nano*ALG/CS-GA_3_ resulted in a 30% increase in fruit fresh weight (Fig. 2d).

The high efficiency of chemical agents delivered using carrier systems has been attributed to greater solubility of the substance, protection against degradation, and prolonged release^2,6,22^. However, other factors are also involved, including the characteristics of the nanoparticles (size, polydispersity index, and zeta potential) and the ways in which they interact with the plant, indicating the importance of evaluating different carrier systems.

An especially interesting feature was that the nanocarrier systems acted to increase the long-term effect of GA_3_ on the development and production of the tomato plants, when the stock or 10-fold dilution formulations were used. It should be noted that the empty nanocarrier systems did not present any effects on fruit production, while loading of GA_3_ in the nanocarriers acted to increase the activity of the plant hormone, according to mechanisms that remain to be elucidated.

### Seed priming to increase tomato productivity

**Figure 3:**
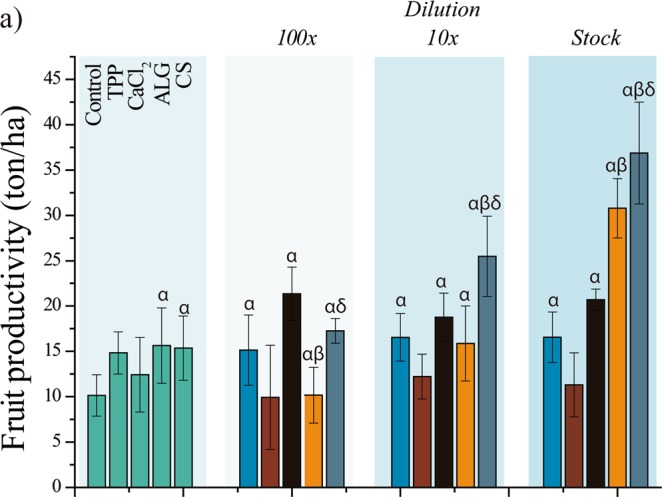
Estimation of plant production, considering the fresh fruit weight (ton) for an area of 10000 m^2^ (1 ha). Seed treatments using the stock solution and dilutions of 1:10 and 1:100 (v/v), equivalent to concentrations of 0.05, 0.005, and 0.0005 mg/mL, respectively. Data presented as means and standard deviations (n = 12). Statistical analyses using one-way ANOVA with Tukey’s post-hoc test (significance level of p < 0.05), where α, β, and δ indicate significant differences relative to the control (water), treatment with GA_3_, and treatment with *nano*CS/TPP-GA_3_, respectively.

The CS nanoparticles showed considerable potential for application in agriculture. The gain provided by nanoencapsulation was evaluated by calculation of the fruit productivity (Fig. 3).

The *nano*ALG/CS-GA_3_ and *nano*CS/TPP-GA_3_ treatments (stock formulations) resulted in productivities of 36.8 ± 5.6 and 30.7 ± 3.2 ton/ha, respectively, compared to 20.7 ± 1.1 ton/ha for GA_3_. All these treatments resulted in productivities that were higher than when the seeds were treated only with water (10.1 ± 2.2 ton/ha).

The productivity gains induced by *nano*ALG/CS-GA_3_ and *nano*CS/TPP-GA_3_ were probably related to the GA_3_ nanoencapsulation, rather than the nanoparticle constituents, given the small or absent effects induced by the treatments with unloaded nanoparticles or the isolated constituents. Even when 10-fold diluted, the *nano*ALG/CS-GA_3_ formulation resulted in a tomato productivity of 25.4 ± 4.4 ton/ha, while GA_3_ led to a productivity of 18.7 ± 2.6 ton/ha. All treatments with GA_3_ resulted in a similar productivity (around 20 ton/ha), even at 100-fold dilution, when GA_3_ treatment was as effective as *nano*ALG/CS-GA_3_ and more effective than *nano*CS/TPP-GA_3_. However, in general, for most of the parameters evaluated, the nanoparticles (especially *nano*ALG/CS-GA_3_) presented greater effects than GA_3_.

It is noteworthy that the effects of *nano*ALG/CS-GA_3_ and *nano*CS/TPP-GA_3_ on tomato productivity were highly dependent on the concentration. The productivity of the plants whose seeds were treated with *nano*ALG/CS-GA_3_ increased from 17.2 to 36.8 ton/ha, when the concentration was increased from the 100-fold dilution to the stock formulation. In the case of the *nano*CS/TPP-GA_3_ treatments, productivity of 30.7 ton/ha was obtained when the stock formulation was used. In contrast, the effect of GA_3_ on fruit productivity was not dependent on the concentration, with values close to 20 ton/ha obtained for all treatments. This fact may explain why the GA_3_ was as efficient as *nano*ALG/CS-GA_3_ and more efficient than *nano*CS/TPP-GA_3_ in increasing fruit productivity, when the 100-fold dilution was used. Nevertheless, for the other concentrations, the *nano*ALG/CS-GA_3_ and *nano*CS/TPP-GA_3_ treatments induced much higher fruit productivities than GA_3_, further evidencing the gains provided by nanoencapsulation.

Seed coating is a methodology used to treat seeds with fungicides and insecticides, in order to protect the seeds in the first stages of development^25^. Theoretical estimation of large-scale productivity indicated that the chitosan nanoparticles containing GA_3_ presented excellent potential for applications in agriculture. The polymers used to manufacture the nanoparticles are very cheap and are essentially produced using water. Moreover, the seed treatment methodology means that the seeds are ready for sowing in the field, while avoiding application of the nanoparticles to the soil and possible contamination of the environment. However, new studies are required to evaluate biosafety issues and to ensure the safe use of this technology.

### Biological efficacy of treatment with the nanoparticles, compared to GA_3_

**Figure 4:**
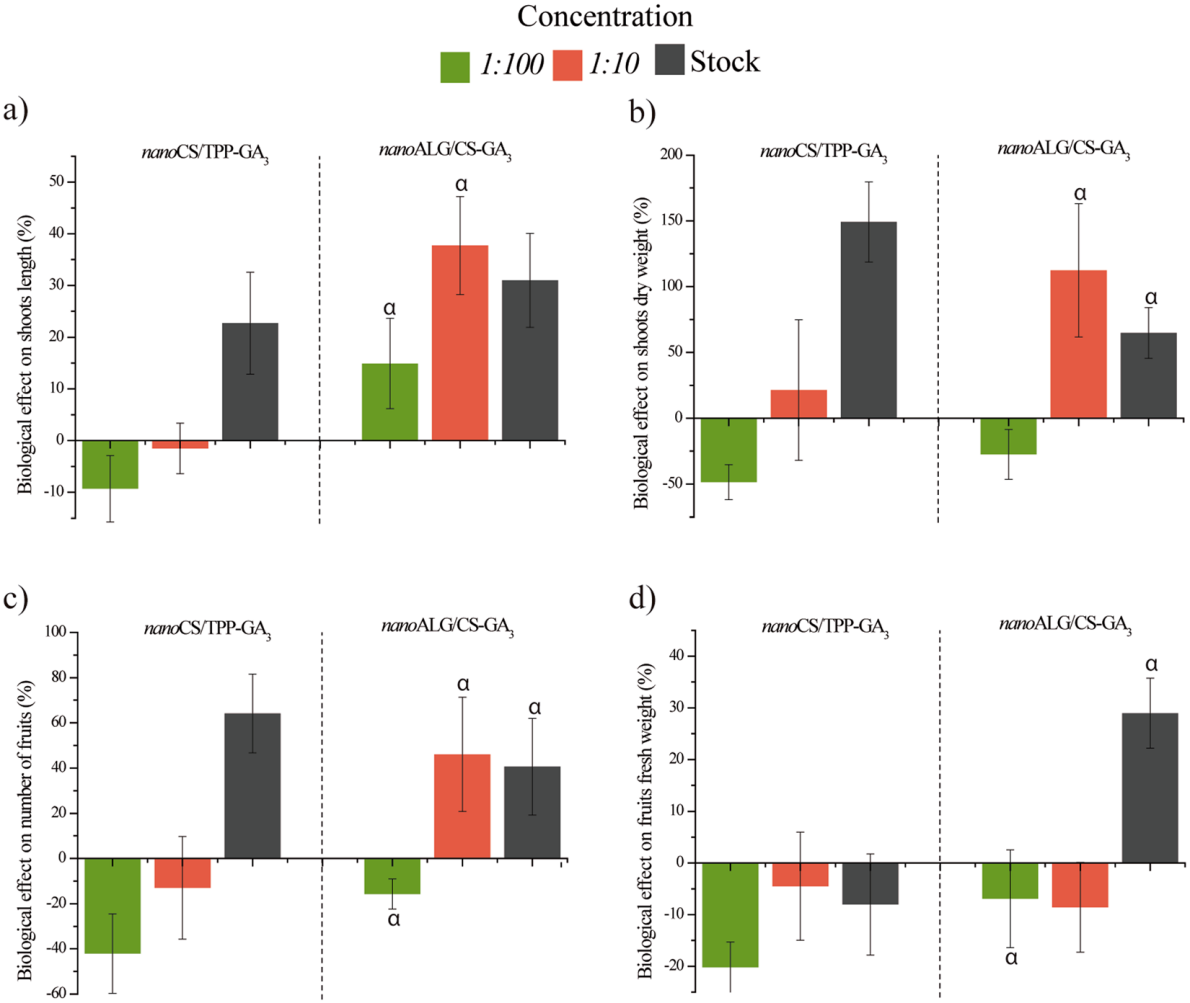
Efficacies of the *nano*ALG/CS-GA_3_ and *nano*CS/TPP-GA_3_ systems, relative to the use of GA_3_, for the different parameters evaluated 120 days after sowing (**a**) length of the aerial part (**b**) dry weight of the aerial part (**c**) number of fruits (**d**) fruit weight. Seed treatments using the stock solution and dilutions of 1:10 and 1:100 (v/v), equivalent to concentrations of 0.05, 0.005, and 0.0005 mg/mL, respectively. Data presented as means and standard deviations (n = 12). Statistical analyses using one-way ANOVA with Tukey’s post-hoc test (significance level of p < 0.05), where α indicates significant difference relative to the treatment with *nano*CS/TPP-GA_3_.

Figure 4 shows the calculated biological efficacies of the different nanocarrier formulations, in comparison with GA_3_, for the parameters evaluated 120 days after sowing.

The greatest effects on shoot length were achieved using *nano*ALG/CS-GA_3_, with increases of 31 and 38% for the stock colloidal dispersion and the 10-fold dilution, relative to the effects of GA_3_ at the same concentrations. For plant dry weight, the biological effect of treatment with *nano*CS/TPP-GA_3_ (stock dispersion) was 149% greater than obtained with GA_3_, while use of *nano*ALG/CS-GA_3_ diluted 10-fold resulted in a dry weight increase of 112%.

The results obtained for plant development (length and dry weight) demonstrated that the nanocarrier systems provided increased efficacy and activity of the plant hormone, compared to GA_3_. Seeds treated with the GA_3_ stock colloidal dispersion and the 10-fold dilution showed no differences compared to the control (water-treated) seeds. However, in the case of the nanocarrier systems, dilution did not reduce the effects, and could even increase them.

The two CS-based nanocarrier systems presented different physico-chemical characteristics, which consequently influenced the release of GA_3_ during the seed priming. *In vitro* assays showed that release from the *nano*ALG/CS-GA_3_ system was initially slower, while almost 100% of the active agent present within the system was released in 24 hours^21^. The *nano*CS/TPP-GA_3_ system presented faster initial release, but 54% of the hormone was retained within the particles after 24 hours. An important consideration is that the conditions of pH and temperature can alter the mechanism and duration of release of GA_3_ ^21^, so further studies are needed to elucidate the nature of release of GA_3_ in plants, as well as to determine how the nanocarrier systems behave within plants.

In terms of fruit production, the *nano*CS/TPP-GA_3_ (stock formulation) system was 64% more effective than GA_3_, while the *nano*ALG/CS-GA_3_ system was 41 and 46% more effective than GA_3_, at dilutions of 10- and 100-fold, respectively. In contrast to *nano*CS/TPP-GA_3_ at the same concentration, the stock colloidal dispersion of the *nano*ALG/CS-GA_3_ formulation was 29% more effective than GA_3_ in increasing fruit weight.

The results described above demonstrated that use of the nanocarrier systems increased the biological activity of GA_3_. Seed priming with the stock formulation and the 10-fold dilution presented long-term effects including enhanced plant development and fruit production. In the case of *nano*ALG/CS-GA_3_, there was also an increase in the weight of the fruits.

In the field, the use of these new technological systems for seed treatment could result in higher agricultural yields. Another feature related to sustainability and food safety is that the treatment was only applied to the seeds, so there was no direct exposure of the formulations to the environment, hence avoiding any possibility of the nanoparticles becoming a source of contamination in water bodies or the soil.

From comparison of the two nanoparticle systems, it could be seen that *nano*ALG/CS-GA_3_ was more effective than *nano*CS/TPP-GA_3_. Consistent with this result, our earlier work showed that *nano*ALG/CS-GA_3_ was more efficient than *nano*CS/TPP-GA_3_ in enhancing the development of apical and lateral roots, as well as the production of photosynthetic pigments, in *Phaseolus vulgaris* plants^21^. The two systems presented different characteristics in terms of particle size, zeta potential, and the mechanism of GA_3_ release, resulting in different biological activities. As an example, the different zeta potentials of the nanocarriers studied (−29 mV for *nano*ALG/CS and +27 mV for *nano*CS/TPP) might have contributed to the observed differences in biological activity. Some studies have shown that the zeta potential has a crucial rule in plant-nanoparticle interaction. Nanoparticles with positive zeta potential have strong interaction with the negative groups of plant cell walls, with low internalization in the cells and a tendency for accumulation on the cell surface. In contrast, nanoparticles with negative zeta potential can be rapidly distributed and internalized into plant cells^39,40^.

Seed priming is a methodology with great potential for use in agriculture. The pretreatment is able to change the metabolism of seeds, not only improving germination and early seedling growth, but also having long-term effects on plant development, with positive impacts on productivity. Many products can be used for seed priming, including plant growth regulators, macro and micro nutrients, and microorganisms. In a recent study, Ma *et al*.^20^ showed that the treatment of *Leymus chinensis* seeds with GA_3_ (non-encapsulated) improved seed germination, plant development, and grass productivity. This growth-promoting effect lasted at least two years, being transmitted to clonal offspring. The authors proposed a transgenerational transmission mechanism for GA_3_ priming effects in the species evaluated. Other studies have demonstrated the beneficial effects of seed priming with GA_3_ on the yields of sunflower and wheat^18,41^. The molecular and physiological mechanisms by which GA_3_ seed priming induces long-term effects in plants are not yet understood, although they may involve epigenetic alterations^20^. Moreover, it remains to be investigated whether exogenous GA_3_ applied during the early development of the embryo could irreversibly alter the transcriptional feedback loops that finely regulate gibberellin homeostasis and signaling^42^.

Here, we observed that GA_3_ nanoencapsulation improved the long-term effect of the seed priming treatment, compared to the use of GA_3_, enhancing the impact of seed priming on tomato productivity. Given the release profiles of *nano*ALG/CS-GA_3_ and *nano*CS/TPP-GA_3_ ^21^, nanoencapsulation did not slow down GA_3_ release to such an extent that would allow delivery of the hormone until the reproductive phase. Hence, a number of different factors could have contributed to the greater development and productivity of the tomato plants following application of CS-based nanoparticles containing GA_3_: (1) The nanoparticles formed a coating on the seeds, which maintained the release of GA_3_ for some period after the seed priming process. The coating of nanoparticles may also have assisted in maintaining seed hydration, which together with the release of GA_3_ had a positive effect on the early development of the seedlings, consequently improving their viability prior to transfer to the soil; (2) The nanoparticles were taken up by the seeds and then continued to release GA_3_ within the plant tissues, hence increasing the endogenous GA_3_ availability more efficiently, compared to the application of GA_3_. The coating of seeds and/or their uptake of nanoparticles provide possible explanations for the positive results obtained using these nanocarrier systems containing GA_3_. However, further studies will be required in order to elucidate their mechanisms of action and the reasons for their effectiveness.

## Conclusions

By using realistic conditions for seed treatment and tomato cultivation, the results indicated that the chitosan-based nanoparticle systems loaded with GA_3_ plant growth regulator presented excellent potential for use in seed treatment in agriculture. The best results were achieved with the *nano*ALG/CS-GA_3_ formulation, which not only enhanced plant development, but also increased fruit productivity. These nontoxic systems deserve further studies aimed at increasing food production in sustainable agricultural practices. Additional ways of applying the systems should be investigated, such as foliar application at different stages of plant development. This is the first work to report the use of these carrier systems for the treatment of seeds, with evaluation of their effects up until production of the final crop. The systems could be used for treating the seeds of different crop species in order to improve productivity, add value to the product, and provide greater profits for the producer. In addition, the encapsulation of other classes of plant growth regulators could also be used to enhance their effectiveness and increase crop productivity. Further studies should be performed to elucidate the ways in which the characteristics of a nanocarrier system influence the biological activities of the active agents.
